# Diabetes mellitus und Straßenverkehr – ein Positionspapier der Österreichischen Diabetesgesellschaft (Update 2023)

**DOI:** 10.1007/s00508-023-02193-5

**Published:** 2023-04-20

**Authors:** Heidemarie Abrahamian, Birgit Salamon, Angelika Lahnsteiner, Christian Schelkshorn, Alexander Bräuer, Lars Stechemesser, Gerd Köhler, Martin Clodi

**Affiliations:** 1 Wissenschaftliches Institut gemäß BundesstatistikG 2008 ÖNACE-CODE: 72.19-0, Privates Institut für Medizin & NLP, Wien, Österreich; 2 KFV (Kuratorium für Verkehrssicherheit), Wien, Österreich; 3 Fachbereich für Biowissenschaften und Medizinische Biologie, Universität Salzburg, Salzburg, Österreich; 4 Erste medizinische Abteilung, Schwerpunkt Stoffwechselmedizin, Landesklinikum Korneuburg/Stockerau, Stockerau, Österreich; 5 Klinik Ottakring, Fünfte Medizinische Abteilung mit Endokrinologie, Rheumatologie und Akutgeriatrie, Wiener Gesundheitsverbund, Wien, Österreich; 6 Landeskrankenhaus, Universitätsklinik für Innere Medizin 1 der PMU, Uniklinikum Salzburg, Salzburg, Österreich; 7 Klinische Abteilung für Endokrinologie und Diabetologie, Medizinische Universität Graz und Rehabilitation für Stoffwechselerkrankungen Aflenz, Graz, Aflenz, Österreich; 8 Krankenhaus der Barmherzigen Brüder Linz und Institut for Cardiovascular and Metabolic Research JKU (ICMR), Johannes Kepler Universität Linz (JKU Linz), Altenberger Straße 69, 4040 Linz, Österreich

**Keywords:** Diabetes mellitus, Führerschein, Fahrsicherheit, Gesetzliche Grundlagen, Hypoglykämien, Hypoglykämiewahrnehmungsstörung, Folgeschäden, Diabetes mellitus, Driving license, Driving safety, Legal regulations, Hypoglycemia, Hypoglycemia unawareness, Late complications

## Abstract

Bei der Beurteilung der gesundheitlichen Eignung zum Lenken eines Kraftfahrzeuges ist die öffentliche Sicherheit (Unfallprävention) das vorrangige Ziel. Der generelle Zugang zu Mobilität sollte jedoch nicht eingeschränkt werden, wenn kein besonderes Risiko für die öffentliche Sicherheit besteht. Für Menschen mit Diabetes mellitus sind im Führerscheingesetz (FSG) und in der Führerscheingesetz-Gesundheitsversorgung (FSG-GV) wichtige Aspekte zur Fahrsicherheit in Zusammenhang mit akuten und chronischen Komplikationen der Erkrankung geregelt. Zu den kritischen Komplikationen, die für die Verkehrssicherheit relevant sind, gehören schwere Hypoglykämie, ausgeprägte Hyperglykämie und Hypoglykämiewahrnehmungsstörung, sowie schwere Retinopathie und Neuropathie, weiters fortgeschrittene Nierenerkrankung und bestimmte kardiovaskuläre Manifestationen. Bei Verdacht auf Präsenz einer dieser Akutkomplikationen oder Folgeschäden ist eine genaue Evaluierung erforderlich.

Darüber hinaus ist die individuelle antihyperglykämische Medikation auf vorhandenes Potenzial für Hypoglykämien zu überprüfen. Sulfonylharnstoffe, Glinide und Insulin gehören in diese Gruppe und sind daher automatisch mit der Auflage einer 5‑jährigen Befristung des Führerscheines assoziiert. Metformin, DPP-4-Hemmer (Dipeptidyl-Peptidase-4-Hemmer, Gliptine), SGLT2-Hemmer (Sodium-dependent-glucose-transporter‑2 inhibitors, Gliflozine), Glitazone und die zu injizierenden GLP-1 Analoga (GLP‑1 Rezeptor Agonisten) weisen kein Hypoglykämiepotential auf und sind daher nicht mit einer Befristung verbunden.

Die FSG-GV gibt Spielraum für Interpretation, sodass im Folgenden spezifische Themen zur Fahrsicherheit für Menschen mit Diabetes mellitus aus fachärztlicher und verkehrsrelevanter Sicht aufgearbeitet wurden. Dieses Positionspapier dient zur Unterstützung von Personen, die mit dieser herausfordernden Materie befasst sind.

## Abstract (en)

Public safety (prevention of accidents) is the primary objective in assessing fitness to drive a motor vehicle. However, general access to mobility should not be restricted if there is no particular risk to public safety. For people with diabetes mellitus, the Führerscheingesetz (Driving Licence Legislation) and the Führerscheingesetz—Gesundheitsverordnung (Driving Licence Legislation Health enactment) regulate important aspects of driving safety in connection with acute and chronic complications of the disease. Critical complications that may be relevant to road safety include severe hypoglycemia, pronounced hyperglycemia and hypoglycemia perception disorder as well as severe retinopathy and neuropathy, endstage renal disease and certain cardiovascular manifestations. If there is a suspicion of the presence of one of these complications, a detailed evaluation is required.

In addition, the individual antihyperglycemic medication should be checked for existing potential for hypoglycemia. Sulfonylureas, glinides and insulin belong to this group and are therefore associated with the requirement of a 5-year limitation of the driver’s license. Other antihyperglycemic drugs without potential for hypoglycemia such as Metformin, SGLT‑2 inhibitors (Sodium-dependent-glucose-transporter‑2 inhibitors, gliflozins), DPP-4-inhibitors (Dipeptidyl-Peptidase inhibitors, gliptins), and GLP‑1 analogues (GLP‑1 rezeptor agonists) are not associated with such a time limitation.

The relevant laws which regulate driving safety give room for interpretation, so that specific topics on driving safety for people with diabetes mellitus are elaborated from a medical and traffic-relevant point of view. This position paper is intended to support people involved in this challenging matter.

## Einleitung

Bei der Beurteilung der gesundheitlichen Eignung zum Lenken eines Kraftfahrzeuges (Kfz) ist die öffentliche Sicherheit (Unfallprävention) das vorrangige Ziel, jedoch sollte der generelle Zugang zu Mobilität nicht eingeschränkt werden, wenn kein besonderes Risiko für die öffentliche Sicherheit besteht.

Nach der österreichischen Straßenverkehrsunfallstatistik sind rund 1 % aller Unfälle auf gesundheitliche Beeinträchtigungen zurückzuführen (Durchschnitt 2017–2021; Statistik Austria). Es ist aber wahrscheinlich, dass in vielen Fällen medizinische Ursachen nicht in der Statistik erfasst werden. Generell liegen wenige Studien zu diesem Thema vor, die Ergebnisse weichen auseinander [1, 2]. In einer schwedischen Studie wurden im Straßenverkehr getötete Pkw-Fahrer obduziert; dabei wurde festgestellt, dass bei 6 % der Fahrer medizinische Faktoren wahrscheinlich der Grund des Unfalls waren, bei 1,3 % war diese Wahrscheinlichkeit hoch [3]. Eine australische Untersuchung kam zu dem Ergebnis, dass 13 % aller untersuchten Unfälle mit Personenschaden und 23 % aller Unfälle mit Getöteten und Schwerverletzten auf medizinische Ursachen zurückzuführen waren [4]. In Finnland, wo alle tödlichen Verkehrsunfälle genau analysiert werden, waren in dem Zeitraum von 2014–2018 16 % dieser Unfälle auf medizinische Ursachen zurückzuführen. Die größte Rolle spielten Herz-Kreislauferkrankungen (relevant bei 13 % aller tödlichen Unfälle), gefolgt von Diabetes mellitus (relevant bei 3 % aller tödlichen Unfälle) [5].

Grundsätzlich kann man davon ausgehen, dass gut eingestellte, stoffwechselstabile und ausreichend geschulte Menschen mit Typ 1 und Typ 2 Diabetes mellitus ein Kfz sowohl der Gruppe 1 als auch der Gruppe 2 sicher lenken können. Dies gilt auch für die Personenbeförderung mittels Taxis, Omnibus und anderen Kfz. Es besteht also grundsätzlich eine Fahreignung.

Unabhängig davon gibt es durch Diabetes mellitus bedingte ungünstige Einflüsse auf das Fahrverhalten, die individuell abzuhandeln sind.

Das Auftreten von schweren Hypoglykämien mit Bewusstseinstrübung oder -verlust ist die häufigste Ursache von Unfällen bei Menschen mit Diabetes mellitus [6].

Daher ist bei einer Behandlung mit Antidiabetika die Hypoglykämien verursachen können, zu erheben, ob und wie oft schwere Hypoglykämien mit Bewusstseinstrübung oder Bewusstseinsverlust auftreten.

Darüber hinaus kann eine Beeinträchtigung der Hypoglykämiewahrnehmung bestehen, die das Spüren von Unterzuckerungen reduziert oder unmöglich macht. Daher stellen Hypoglykämiewahrnehmungsstörungen im Straßenverkehr eine Gefahr dar. Auch Hyperglykämien können ab einer bestimmten Höhe einen ungünstigen Einfluss auf das Fahrverhalten nehmen [7]. Aus den genannten Gründen sind im Rahmen der Anamnese neben schweren Hypoglykämien auch Hyperglykämien, sowie das Bestehen einer Hypoglykämiewahrnehmungsstörung zu erfassen.

Weiters können auch Spätfolgen des Diabetes mellitus in fortgeschrittener Form zur Einschränkung der Fahrtauglichkeit führen. Dazu zählen vor allem diabetische Retinopathie, bestimmte Formen der diabetischen Neuropathie sowie zerebro- und kardiovaskuläre Erkrankungen [8].

Die Häufigkeit von Kfz-Unfällen bei Personen mit und ohne Diabetes mellitus wurde in einer Metaanalyse untersucht. Dabei zeigte sich für Personen mit Diabetes ein relatives Risiko für einen Unfall im Vergleich zu Personen ohne Diabetes von 1,12–1,19, also ein um 12–19 % erhöhtes Risiko [9–12]. Dieses gering erhöhte Risiko ist im Vergleich zu anderen Risikogruppen zu betrachten. Menschen mit Schlafapnoe-Syndrom weisen ein um ca. 140 % erhöhtes Risiko und Menschen mit ADHS (Aufmerksamkeits Defizit Hyperaktivitätsstörung) ein um ca. 340 % erhöhtes Risiko für Kfz-Unfälle auf (Tab. 1; [11, 13, 14]).

**Table 1:** 

DIAGNOSE	RELATIVES RISIKO
Diabetes mellitus	1,12–1,19
Aufmerksamkeits-Defizit/Hyperaktivitätsstörung	Ca. 4,0
Schlafapnoe-Syndrom	Ca. 2,4

Eine zumindest vorübergehende Entziehung der Lenkberechtigung kann erforderlich sein, wenn die Voraussetzungen für die gesundheitliche Eignung zum Lenken eines Kfz nicht erfüllt werden, nämlich bei nicht kontrollierten Nebenwirkungen der Behandlung oder bestimmten Komplikationen der Erkrankung. Zu bedenken ist, dass eine Entziehung der Lenkberechtigung eine Benachteiligung des Betroffenen in vielen sozialen Bereichen darstellt. Generell ist es sinnvoll, Hochrisikogruppen herauszufiltern, eine entsprechende Bewertung bezüglich Selbst- bzw Fremdgefährdung durch Teilnahme am Straßenverkehr zu erstellen und bei Erfordernis bestimmte Auflagen zu erteilen.

## Rechtliche Grundlagen

Grundlage für die gesetzlichen Regelungen über die gesundheitliche Eignung ist die EU-Führerscheinrichtlinie 2006/126/EG. In deren Anhang III sind die Anforderungen für das Erteilen einer Lenkberechtigung bei Vorliegen verschiedener Krankheiten im Detail geregelt, darunter auch Diabetes mellitus.

In Österreich wurden die Vorgaben der Richtlinie im Führerscheingesetz (FSG, BGBl I 1997/120) und in der Führerscheingesetz-Gesundheitsverordnung (FSG-GV, BGBl II 1997/322) umgesetzt. Aus Sicht der Verordnung gilt eine Person als zum Lenken von Kraftfahrzeugen hinreichend geeignet, wenn keine der folgenden Krankheiten festgestellt wurde (§ 5 Abs. 1 FSG-GV):

1. Schwere Allgemeinerkrankungen oder schwere lokale Erkrankungen, die das sichere Beherrschen des Kraftfahrzeuges und das Einhalten der für das Lenken des Kraftfahrzeuges geltenden Vorschriften beeinträchtigen könnten.
2. Organische Erkrankungen des zentralen oder peripheren Nervensystems, die das sichere Beherrschen des Kraftfahrzeuges und das Einhalten der für das Lenken des Kraftfahrzeuges geltenden Vorschriften beeinträchtigen könnten.
3. Erkrankungen, bei denen es zu unvorhersehbaren Bewusstseinsstörungen oder -trübungen kommt.
4. Schwere psychische Erkrankungen gemäß § 13 FSG-GV sowie
5. Alkoholabhängigkeit oder
6. andere Abhängigkeiten, die das sichere Beherrschen des Kraftfahrzeuges und das Einhalten der für das Lenken des Kraftfahrzeuges geltenden Vorschriften beeinträchtigen könnten.
7. Augenerkrankungen, die das Sehvermögen beeinträchtigen.

Somit werden gemäß § 5 Abs. 3 FSG-GV Erkrankungen, die zu Bewusstseinsstörungen oder -verlust führen können, als erhebliches Gefahrenpotenzial bewertet. Bei Diabetes mellitus kann unter Therapie mit bestimmten blutzuckersenkenden Medikamenten und unter besonderen sonstigen Voraussetzungen eine schwere Hypoglykämie mit Bewusstseinsstörung oder -verlust auftreten. Auch Augenerkrankungen, die das Sehvermögen beeinträchtigen, und organische Erkrankungen des zentralen und peripheren Nervensystems können das sichere Beherrschen eines Kraftfahrzeuges im Sinne von § 5 einschränken.

Nähere Bestimmungen zu Diabetes („Zuckerkrankheit“) sind in den 4 Absätzen des § 11 FSG-GV geregelt:

1. *Zuckerkranken darf* *eine Lenkberechtigung nur nach einer befürwortenden fachärztlichen Stellungnahme erteilt oder belassen werden, aus der insbesondere auch hervorgeht, dass der Zuckerkranke die mit Hypoglykämie verbundenen Risiken versteht und seinen Zustand angemessen beherrscht.*
2. *Zuckerkranken, die mit Insulin oder bestimmten Tabletten behandelt werden müssen, darf eine Lenkberechtigung der Gruppe 1 nur für einen Zeitraum von höchstens fünf Jahren unter der Auflage ärztlicher Kontrolluntersuchungen und amtsärztlicher Nachuntersuchungen erteilt oder belassen werden.*
3. *Zuckerkranken, die mit Insulin oder bestimmten Tabletten behandelt werden müssen, darf eine Lenkberechtigung der Gruppe 2 nur für einen Zeitraum von höchstens drei Jahren unter der Auflage ärztlicher Kontrolluntersuchungen und amtsärztlicher Nachuntersuchungen und unter Einhaltung folgender Voraussetzungen erteilt oder belassen werden:*
  - *der Lenker gibt eine Erklärung ab, dass in den letzten 12 Monaten keine Hypoglykämie aufgetreten ist, die eine Hilfe durch eine andere Person erforderlich macht (schwere Hypoglykämie);*
  - *es besteht keine Hypoglykämie-Wahrnehmungsstörung;*
  - *der Lenker weist eine angemessene Überwachung der Krankheit durch regelmäßige Blutzuckertests nach, die mindestens zweimal täglich sowie zu jenen Zeiten vorgenommen werden, zu denen die Person üblicherweise Kraftfahrzeuge lenkt;*
  - *der Lenker zeigt, dass er die mit Hypoglykämie verbundenen Risiken versteht;*
  - *es liegen keine anderen Komplikationen der Zuckerkrankheit vor, die das Lenken von Fahrzeugen ausschließen.*
4. *Zuckerkranken, bei denen innerhalb von 12 Monaten zwei Mal eine Hypoglykämie aufgetreten ist, die eine Hilfe durch eine andere Person erforderlich macht (wiederholte schwere Hypoglykämien), sowie Zuckerkranken, die an Hypoglykämie-Wahrnehmungsstörung leiden, darf eine Lenkberechtigung nur nach einer befürwortenden fachärztlichen Stellungnahme sowie unter der Auflage von Kontrolluntersuchungen und Nachuntersuchungen erteilt oder belassen werden. Bei wiederholten schweren Hypoglykämien im Wachzustand darf eine Lenkberechtigung erst drei Monate nach der letzten Episode erteilt oder verlängert werden.*

Abs. 1 und 4 gelten sowohl für Lenker der Gruppe 1 als auch für Lenker der Gruppe 2, Abs. 2 nur für Lenker der Gruppe 1 und Abs. 3 nur für Lenker der Gruppe 2.

Die Unterscheidung in Gruppe 1 und Gruppe 2 ergibt sich aus der Unterteilung der Kraftfahrzeuge in Klassen, wobei mehrere Klassen zu einer Gruppe zusammengefasst werden. Gruppe 1 umfasst Pkw sowie Mopeds und Motorräder (Führerscheinklassen A, A1, A2, AM, B, BE), Gruppe 2 Lkw und Busse (Führerscheinklassen C, CE, C1, C1E, D, DE, D1, D1E). Für die beiden Gruppen gibt es unterschiedliche Vorgaben für Menschen mit Diabetes, wobei die Vorgaben für Gruppe 2 deutlich strenger sind. Bei Gruppe 2‑Lenkern, die mit Insulin oder oralen Antidiabetika mit Hypoglykämierisiko behandelt werden, darf innerhalb der letzten 12 Monate keine schwere Hypoglykämie aufgetreten sein; bei Gruppe 1‑Lenkern ist eine wiederholte schwere Hypoglykämie ein Ausschlussgrund. Eine Befristung erfolgt bei Gruppe 2 auf höchstens drei Jahre, bei Gruppe 1 auf höchstens fünf Jahre.

## Verkehrsrelevante akute Komplikationen des Diabetes mellitus

### Hypoglykämien

Bezüglich Diabetes mellitus wird im EU-Report (*Diabetes and Driving in Europe*) die schwere Hypoglykämie mit Bewusstseinsbeeinträchtigung als elementares Ereignis dargestellt, das für die Beschränkung des Lenkens eines Kraftfahrzeuges relevant ist [15].

Die Beeinträchtigung des Bewusstseins bei Hypoglykämie erklärt sich dadurch, dass das Gehirn hauptsächlich Glukose als Energiequelle verwendet. Wenn der Blutzucker unter 60 mg/dl fällt, entwickeln sich Symptome der Neuroglukopenie wie kognitive Störungen, die die Fahrtüchtigkeit beeinträchtigen können. Adrenerge Symptome beginnen jedoch in der Regel bei höheren Glukosewerten, so dass der Patient Zeit hat, auf diese Warnungen zu reagieren und Kohlenhydrate zu sich zu nehmen, um den Blutzucker anzuheben.

Für Fahrzeuglenker mit Diabetes mellitus, die mit blutzuckersenkenden Medikamenten behandelt werden, die zu einer schweren Hypoglykämie führen können, wie Sulfonylharnstoffe, Glinide und Insulin, ist eine besondere Achtsamkeit in Hinblick auf derartige Ereignisse geboten. Sofern eine Schulung für das korrekte Verhalten bei Auftreten von Symptomen der Hypoglykämie absolviert wurde und die Symptome einer Hypoglykämie auch rechtzeitig erkannt und zugeordnet werden können, sodass wirksame Gegenmaßnahmen ergriffen werden können, besteht kein Grund, eine Lenkberechtigung zu entziehen oder vorzuenthalten. Kritisch wird es, wenn Kraftfahrzeuglenker die Symptome der Hypoglykämie nicht ausreichend gut oder gar nicht wahrnehmen. Dann besteht eine Hypoglykämiewahrnehmungsstörung, die aufgrund der progredienten Neuroglukopenie mit erheblichen Gefahren im Verkehr assoziiert ist.

Die Definition der Hypoglykämie ist generell keine einheitliche, sodass bei Fragen zur Eignung ein Kfz zu lenken, zu berücksichtigen ist, bei welchen Glukosewerten bereits kognitive Beeinträchtigungen und Defizite in der Reaktionskette auftreten. Laut internationaler Definition ist ein Glukosewert von unter 70 mg/dl als Level 1 Hypoglykämie definiert, eine kognitive Beeinträchtigung ist möglich jedoch individuell unterschiedlich. Daher ist ein Glukosewert knapp darüber als Interventionsschwelle für das Zuführen von Kohlenhydraten zu betrachten [8]. Bei Hypoglykämien kommt es auch zur Verschlechterung der visuellen Wahrnehmung. Daher wird empfohlen bei grenzwertig niedrigen Glukosewerten bereits vor Fahrtantritt und während der Fahrt einen Kohlenhydrat-Snack zu sich zu nehmen [8, 11].

Bei Gruppe-1-Lenkern muss die Lenkberechtigung bei Auftreten von zwei schweren Hypoglykämien innerhalb des letzten Jahres, die Fremdhilfe erforderten, vorübergehend entzogen werden; bei Gruppe 2‑Lenkern bereits ab der ersten schweren Hypoglykämie innerhalb der letzten 12 Monate. Erst wenn durch Schulung, Therapieumstellung oder Einsatz neuer Technologien angenommen werden kann, dass Symptome der Hypoglykämie wieder rechtzeitig erkannt werden, kann die Lenkberechtigung unter Auflagen erneut erteilt werden. Der Einsatz eines Glukosesensors mit Hypoglykämiewarnung oder eines Hybrid Closed Loop Systems bei einer Insulinpumpentherapie sowie die Umstellung auf Antidiabetika ohne Hypoglykämierisiko sind Optionen zur Wiederherstellung der gesundheitlichen Eignung. Generell empfiehlt sich in diesen Fällen eine Befristung auf ein Jahr, mit Überprüfung der Umsetzung der Auflagen bzw des Wiedererlangens der Hypoglykämiewahrnehmung. Bei wiederholten schweren Hypoglykämien im Wachzustand darf erst drei Monate nach der letzten Episode wieder eine Lenkberechtigung erteilt werden.

Für Kraftfahrzeuge der Gruppe 2, mit denen Personen befördert werden, also Autobusse (Klasse D), sollte die Erteilung einer Lenkberechtigung bei Therapien mit Hypoglykämierisiko unter Bedachtnahme auf die besonderen Anforderungen erfolgen. Zu bedenken ist, dass es für den Buslenker realistisch nicht möglich ist, die Fahrt eines Linienbusses zu unterbrechen, um den Blutzucker zu messen oder bei Hypoglykämie Kohlenhydrate zuzuführen und daraufhin zumindest 15 min abzuwarten, bevor die Fahrt fortgesetzt werden kann. Umso mehr muss das Auftreten von Hypoglykämien in dieser Personengruppe vermieden werden. Auch in dieser Gruppe kann abhängig vom Diabetestyp durch Einsatz eines Glukosesensors mit Hypoglykämiewarnung oder Hybrid Closed Loop Systemen bei einer Insulinpumpentherapie sowie durch die Umstellung auf Antidiabetika ohne Hypoglykämierisiko die gesundheitliche Eignung hergestellt werden.

Auch für das Lenken von Einsatzfahrzeugen wie Rettungswagen stellt eine Hypoglykämie „am Steuer“ eine ungünstige Situation dar, insbesondere da durch den Zeitverzug bei Hypoglykämie-Intervention Schaden für den dringend zu transportierenden Patienten entstehen kann. Auch in dieser Gruppe sollte obige Forderung des Vermeidens von Hypoglykämien umgesetzt werden. Rechtlich gelten derzeit für Rettungsfahrzeuge bis 5500 kg, die mit einem Führerschein der Klasse B allein oder in Verbindung mit einer speziellen, von der Rettungsorganisation ausgestellten Berechtigung (§ 1 Abs. 3 4. Satz FSG) gelenkt werden, nur die Anforderungen für Gruppe-1-Lenker.

Taxilenker benötigen einen Führerschein der Klasse B; es gelten daher in Österreich die Voraussetzungen für Gruppe-1-Lenker. Die EU-Führerscheinrichtlinie enthält eine Bestimmung, dass auf Lenker der Klasse B, die ihren Führerschein für berufliche Zwecke verwenden (Taxis, Krankenwagen usw.), die Bestimmungen für Lenker der Gruppe 2 angewandt werden können. Diese Möglichkeit wurde jedoch in Österreich nicht umgesetzt, obwohl der EU-Report dies empfiehlt [15].

### Hypoglykämiewahrnehmungsstörung

Die Hypoglykämiewahrnehmungsstörung wird mit einer Häufigkeit von 25 % bei Menschen mit Typ 1 Diabetes mellitus und von 10 % bei Menschen mit Typ 2 Diabetes mellitus angegeben, d. h. jede vierte Person mit Typ 1 Diabetes mellitus und jede zehnte Person mit Typ 2 Diabetes mellitus ist betroffen [16, 17].

Menschen mit einer Hypoglykämiewahrnehmungsstörung erkennen aufgrund einer reduzierten oder fehlenden Antwort der gegenregulatorischen Hormone nicht, dass der Blutzuckerspiegel absinkt. Kritisch ist ein Absinken unter die Schwelle für Neuroglukopenie die bei Typ 1 Diabetes in etwa bei 54 mg/dl liegt [18]. In diesem Bereich und darunter kommt es zu schwerwiegenden kognitiven Ausfällen und in weiterer Folge zu Bewusstseinstrübung bis zum Bewusstseinsverlust. Das Risiko für eine schwere Hypoglykämie ist ca. 10-mal höher als das von Menschen mit Diabetes ohne diese Störung. Die Hypoglykämiewahrnehmungsstörung kann in unterschiedlicher Ausprägung auftreten, von einer geringen Wahrnehmungsstörung bis zum kompletten Fehlen von adrenergen Warnsymptomen. Aufgrund dieser Breite wurde eine Einteilung in partielle/reversible und irreversible Hypoglykämiewahrnehmungsstörung getroffen [19–21].

Die Beurteilung, ob eine Hypoglykämiewahrnehmungsstörung vorliegt, ist schwierig, da die Selbsteinschätzung der Patienten eingeschränkt ist. In einer Studie von Cox et al., wurden 37 Studienteilnehmer mit Diabetes mellitus Typ 1 während progressiver Hypoglykämie einer Fahrsimulation unterzogen und sollten ihre Fahrtauglichkeit bei drei unterschiedlichen Blutzuckerbereichen bewerten [22]. Während aller drei hypoglykämischer Blutzucker-Bereiche war das Fahren signifikant beeinträchtigt, und die Probanden waren sich ihrer Beeinträchtigung des Fahrens bewusst. Die Entscheidung für Korrekturmaßnahmen wurde jedoch erst bei einem Blutzucker von < 50 mg/dl getroffen.

Bei Vorliegen einer Hypoglykämiewahrnehmungsstörung ist wahrscheinlich von einer erheblichen Fremd- und Selbstgefährdung im Straßenverkehr auszugehen. Allerdings gibt es keine Evidenz für einen gesicherten Zusammenhang zwischen Hypoglykämiewahrnehmungsstörung und Häufigkeit von Autounfällen [6, 9].

Das Erheben einer genauen Anamnese, die diese Störung erfasst, ist unumgänglich. Die Anamnese beschränkt sich nicht allein auf die Frage, ob eine Hypoglykämiewahrnehmungsstörung vorliegt, sondern sollte auch Fragen beinhalten wie: „Bei welchem Blutzuckerwert fühlen Sie sich noch fit?“ bzw. „Was war der tiefste je von Ihnen gemessene Blutzuckerwert, bei dem Sie sich noch fit fühlten?“ Da die Möglichkeit besteht, dass den Betroffenen die Hypoglykämiewahrnehmungsstörung gar nicht bewusst ist, können durch anamnestische Fragen wie den beiden letzten, konkrete Informationen eingeholt werden. Die Anwendung des Clarke-Scores ist ebenso eine Möglichkeit eine gestörte Wahrnehmung von Hypoglykämien zu diagnostizieren (siehe später im Text) [23].

### Maßnahmen zur Abwendung von schweren Hypoglykämien

Zur Abwendung von schweren Hypoglykämien sind mehrere wirksame Maßnahmen verfügbar:

1. **Wahl einer Therapieform** die möglichst wenig Hypoglykämierisiko aufweist (siehe Kapitel Medikamente).
2. **Kontinuierliche Glukosemessung mit Warnfunktion**
  Das kontinuierliche Glukosemonitoring (rtCGM) zeichnet sich durch fortlaufende Messung von Gewebsglukosewerten mit Hilfe eines Sensors aus, verbunden mit einer Warnung vor Hypoglykämien ab definierten Schwellenwerten. In der INCONTROL-Studie, einer randomisierten, cross-over Studie mit 52 Typ 1 Diabetes Patienten mit einer eingeschränkten Hypoglykämiewahrnehmung konnte durch Anwendung von CGM im Vergleich zur üblichen Blutglukoseselbstkontrolle die Anzahl schwerer Hypoglykämien signifikant reduziert werden [24].
3. **Sensorgestützte Insulinpumpentherapie**
  Die sensorgestützte Insulinpumpentherapie zeichnet sich durch die Verbindung eines Glukosesensors mit einer Algorithmus gesteuerten Insulinpumpe aus. Bei älteren Systemen erfolgt lediglich eine automatische Unterbrechung der Insulinzufuhr, wenn eine Hypoglykämie droht. Hybrid Closed loop Systeme steuern zusätzlich die Basalinsulinzufuhr individuell und führen auch automatische Korrekturen bei zu hohen Glukosewerten durch. Mahlzeitenbezogene Insulinboli müssen weiterhin manuell abgegeben werden. Es sind Systeme verfügbar bei denen der Algorithmus in die Pumpe integriert ist, aber auch Systeme, welche über eine App gesteuert werden. Studien mit solchen Systemen zeigen eine verbesserte Stoffwechselkontrolle mit weniger Hyper- und Hypoglykämien [25]. Mit Hilfe dieser Systeme ist es möglich eine signifikant längere Zeit im Glukosezielbereich (Time in Range TIR) zu erreichen [26].
4. **Hypoglykämiewahrnehmungstraining**
  Dieses Training ist ein psychoedukativer Prozess, in dem die verschiedenen Aspekte der Hypoglykämie beleuchtet und den betroffenen Personen nahegebracht werden. Weiters wird durch die sorgfältige Vermeidung von Hypoglykämien für 2‑3 Wochen die gegenregulatorische Kompetenz des Körpers in vielen Fällen wiederhergestellt bzw verbessert. Die Zielwerte für die Glukose werden während dieser Periode höher angesetzt [27].

### Hyperglykämien

Inwiefern hyperglykämische Zuckerwerte ab einer bestimmten Höhe zu Beeinträchtigungen des Lenkverhaltens führen können, ist explizit nicht untersucht. In einigen Studien konnten Auswirkungen der Hyperglykämie auf die Konzentrationsfähigkeit und Schnelligkeit der Informationsverarbeitung nachgewiesen werden [28, 29]. Eine Studie an Schulkindern mit Diabetes mellitus Typ 1 zeigte, dass die Auswirkungen auf die Ergebnisse kognitiver Tests vergleichbar waren bei Blutzuckerwerten von 54 mg/dl und 400 mg/dl, also sowohl starke Unterzuckerung als auch starke Blutzuckererhöhung zu erheblichen Beeinträchtigungen der kognitiven Leistung führten [30, 31]. Ein direkter Zusammenhang zwischen Hyperglykämie und Häufigkeit von Unfällen ist nicht dokumentiert, diesbezüglich liegen auch keine Studien vor.

Bei starker hyperglykämischer Entgleisung mit nachhaltigen Auswirkungen auf den Stoffwechsel wie Elektrolytverschiebungen, Veränderungen im Säure-Basenhaushalt und in der Osmolarität ist anzunehmen, dass auch eine ungünstige Beeinflussung des Lenkverhaltens auftritt.

## Verkehrsrelevante Spätfolgen des Diabetes mellitus

Inwieweit vorhandene Spätfolgen des Diabetes mellitus die gesundheitliche Eignung zum Lenken eines Kfz beeinträchtigen, ist individuell zu prüfen.

### Diabetes mellitus und Augen

Einschränkungen des Sehvermögens im Rahmen einer proliferativen diabetischen Retinopathie, einer Makulopathie, eines Kataraktes und in selteneren Fällen einer diabetischen Optikusneuropathie können sich verkehrsrelevant auswirken. Auch zumeist reversible Augenmuskellähmungen können auftreten, und zu einer vorübergehenden Einschränkung der gesundheitlichen Eignung führen.

Daher sind Augenuntersuchungen in regelmäßigen Abständen erforderlich. Der fachärztliche Befund des Augenarztes umfasst neben der Erfassung der Sehschärfe insbesondere die Beurteilung des Augenhintergrundes, eine Gesichtsfelduntersuchung und zusätzlich die Prüfung des Dämmerungssehens mit Angabe der Kontraststufe. (siehe Leitlinien für die gesundheitliche Eignung von Kraftfahrzeuglenkern; Abschn. 3.4 Mängel des Sehvermögens [32]).

### Diabetes mellitus und Niere

Die Nephropathie zeigt erst in einem sehr fortgeschrittenen Stadium signifikante Auswirkungen auf die Fahrtüchtigkeit. Durch die Bestimmung von Serum-Kreatinin, dem Stadium der Albuminurie und der glomerulären Filtrationsrate kann das Stadium der Nierenfunktionsstörung beurteilt werden. Bei fortgeschrittener Nierenerkrankung sind ärztliche Kontrolluntersuchungen im Rahmen von Befristungen in Erwägung zu ziehen. (siehe Leitlinien für die gesundheitliche Eignung von Kraftfahrzeuglenkern; Abschn. 3.11 Nierenerkrankungen [32]).

### Diabetes mellitus und Nervensystem

Die periphere diabetische Polyneuropathie, insbesondere die Schädigung der sensiblen Nervenfasern mit Einschränkung des Berührungsempfindens, kann zu verkehrsrelevanten Beeinträchtigungen führen, sodass sich daraus Konsequenzen für die gesundheitliche Eignung ergeben können. Ein direkter Zusammenhang zwischen Unfallhäufigkeit und Neuropathie ist nicht dokumentiert. Die Untersuchung mit Monofilament und Stimmgabel gibt eine orientierende Information zur Neuropathie. (siehe Kapitel: Diabetische Neuropathie und diabetischer Fuß. ÖDG Leitlinie 2023) Eine relativ neue Studie zu Neuropathie und Fahrverhalten zeigt bei einer kleinen Fallzahl eine signifikante Verlängerung der Bremspedal-Bedienungszeit bei Probanden mit diabetischer Neuropathie im Vergleich zu Probanden mit Diabetes und ohne Neuropathie [33, 34] (siehe Leitlinien für die gesundheitliche Eignung von Kraftfahrzeuglenkern; Abschn. 3.8.4 Diverse Erkrankungen des Nervensystems [32]).

### Diabetes mellitus und kardiovaskuläre Erkrankungen

Bei Menschen mit Diabetes treten kardiovaskuläre Erkrankungen häufiger und früher auf als in der gesunden Bevölkerung. Auswirkungen von makrovaskulären Spätfolgen wie koronare Herzkrankheit, zerebrovaskuläre Erkrankung und periphere arterielle Verschlusskrankheit auf die gesundheitliche Eignung sind individuell zu beurteilen. Insgesamt ist die Frequenz von Verkehrsunfällen, die durch kardiovaskuläre Erkrankungen auftreten gering [1] (siehe Leitlinien für die gesundheitliche Eignung von Kraftfahrzeuglenkern; Abschn. 3.6. Herz-Kreislauf-Erkrankungen [32]). Zu beachten ist weiters, dass Hypoglykämien das Risiko für das Auftreten von kardiovaskulären Ereignissen erhöhen können [35].

Risikofaktoren, die das Entstehen von atherosklerotischen Ereignissen begünstigen, wie Hypertonie, Hyperlipidämie und Glykämie sind laut Leitlinien der Österreichischen Diabetesgesellschaft zu therapieren, um Inzidenz und Progredienz von kardiovaskulären Ereignissen hintanzuhalten [36].

Eine Herzinsuffizienz kann bei 10–20 % der Menschen mit Typ-2-Diabetes nachgewiesen werden. Relevant für die gesundheitliche Eignung ist eine Herzinsuffizienz mit NYHA Stadium III–IV.

### Diabetes mellitus und Schlafapnoe-Syndrom

Schlafapnoe tritt bei Menschen mit Diabetes mellitus gehäuft auf (23–58 %) und ist als verkehrsrelevant einzustufen [11, 14]. Bei anamnestisch erhöhter Tagesschläfrigkeit sollte eine gezielte Diagnostik initiiert werden. Die bestätigte Diagnose der Schlafapnoe steigert das Risiko für Verkehrsunfälle um das 1,2–4,9-fache [14]. Zur gesundheitlichen Eignung beim obstruktiven Schlafapnoe-Syndrom wurden im Jahr 2014 in der EU-Führerscheinrichtlinie Vorgaben geschaffen, die vorsehen, dass bei Personen mit Verdacht auf ein mittelschweres oder schweres obstruktives Schlafapnoe-Syndrom, eine Lenkberechtigung nur nach Einholung einer fachärztlichen Stellungnahme erteilt oder belassen werden kann (umgesetzt in § 12b FSG-GV; s. Leitlinien für die gesundheitliche Eignung von Kraftfahrzeuglenkern; Abschn. 3.2 Obstruktives Schlafapnoesyndrom [32]).

## Medikamente zur Blutzuckersenkung – Typ 1 und Typ 2 Diabetes mellitus

Exogenes Insulin und bestimmte blutzuckersenkende Medikamente mit insulinotroper Wirkung können zu Hypoglykämien führen und damit Einfluss auf die Verkehrstüchtigkeit nehmen. Zu diesen Medikamenten, die ein substanzeigenes Hypoglykämierisiko aufweisen, zählen Sulfonylharnstoffe, Glinide und Insulin. Eine Aufklärung der Patient:innen über das mögliche Auftreten von Hypoglykämien ist bei Initiierung dieser Therapien erforderlich, ebenso sind Informationen über damit verbundene mögliche Gefahren im Straßenverkehr zu vermitteln.

Metformin, DPP-4-Hemmer (Dipeptidyl-Peptidase-4-Hemmer, Gliptine), SGLT2-Hemmer (Sodium-dependent-glucose-transporter‑2 inhibitors, Gliflozine), Glitazone und die zu injizierenden GLP-1 Analoga (GLP‑1 Rezeptor Agonisten) weisen kein substanzeigenes Hypoglykämie-Risiko auf. Auch die in Kürze zur Verfügung stehenden dualen GLP-1/GIP Agonisten (Glucose-dependent insulinotropic Peptides (GIP) kombiniert mit einem GLP‑1 Analogon), verfügen über kein substanzeigenes Hypoglykämiepotential.

### Besonderheiten bei Typ 1 DM

Basalinsuline der ersten Generation (NPH-Insulin) sollten aufgrund der erhöhten Hypoglykämiegefahr bei Menschen mit Diabetes mellitus Typ 1 nicht mehr angewendet werden. Basalinsuline der zweiten Generation (Insulin glargin, Insulin detemir) haben gegenüber NPH-Insulin schon ein verbessertes Wirkprofil sollten aber auch nur noch in speziellen Situationen zur Anwendung kommen, da auch diese gegenüber den Insulinen der 3. Generation (Insulin degludec, Insulin glargin U300) ein erhöhtes Hypoglykämierisiko aufweisen [37]. Bezüglich der kurzwirkenden Insuline gibt es aktuell keine Evidenz, die einem der derzeit in Verwendung befindlichen Analoga einen Vorteil zuspricht.

Neue Technologien wie Glukosesensoren und sensorgestützte Insulinpumpentherapien reduzieren das Auftreten von Hypoglykämien. Unabhängig davon, welche Therapie oder welche technischen Hilfsmittel angewendet werden, ersetzen diese aber nicht eine konsequente und genaue Schulung betreffend Verhaltensregeln beim Lenken eines KFZ.

### Besonderheiten bei Typ 2 DM

Personen mit Typ 2 Diabetes mellitus stellen eine heterogene Gruppe von Patient:innen dar. Einerseits sind es jüngere Menschen, die wenig bis keine antidiabetische Medikation brauchen (vor allem nur Pharmaka mit geringem bis keinem Hypoglykämierisiko), mit wenigen Spätfolgen und andererseits ältere bis alte Patient:innen, mit multiplen Komorbiditäten und Spätfolgen sowie einer deutlichen Polypharmazie.

Ma et al. konnten an einer kleinen Gruppe von Männern mit Typ 2 Diabetes mellitus (Alter 52,7 Jahre) zeigen, dass im Vergleich zu Probanden ohne Diabetes deutlich schlechtere Fahrleistungen (Spurhaltung, Reaktionszeit) erbracht wurden. Interessanterweise waren in der diabetischen Population selbstberichtete Hypoglykämien in der Anamnese (ohne Bedarf einer Fremdhilfe) mit einer besseren Fahrleistung assoziiert [38].

Für ältere Menschen (65–79 Jahre) konnten Hill et al. im Rahmen der LongROAD Study (Longitudinal Research on Aging Drivers) zeigen, dass einerseits in dieser Gruppe eine deutliche Polypharmazie vorlag (Median 7 Medikamente/Teilnehmer:innen) und andererseits ein ungünstiger Zusammenhang zwischen bestimmten Medikamenten und dem Fahrverhalten in Bezug auf Beschleunigen und plötzliches Abbremsen bestand [39]. Eine spezielle Auswertung für die Gruppe mit Diabetes bezüglich antidiabetischer Medikation wurde nicht durchgeführt.

Insgesamt ist die Datenlage bezüglich Auswirkungen der Diabetesmedikation auf die Sicherheit beim Lenken eines Kfz bei Menschen mit Typ 2 Diabetes mellitus nicht ausreichend, um etwaige Schlussfolgerungen zu ziehen.

## Publizierte nationale und internationale Richt- bzw Leitlinien

Einige publizierte Richt- und Leitlinien sollen in diesem Kapitel kurz vorgestellt und kommentiert werden.

In Österreich wurden im Auftrag des Bundesministeriums für Verkehr, Innovation und Technologie (jetzt: Bundesministerium für Klimaschutz, Umwelt, Energie, Mobilität, Innovation und Technologie, BMK) unter der Leitung des Kuratoriums für Verkehrssicherheit (KFV) und in Zusammenarbeit mit einer Expertengruppe **„Leitlinien für die gesundheitliche Eignung von Kraftfahrzeuglenkern“, (Ein Handbuch für Amts- und Fachärzte und die Verwaltung), **erarbeitet [32]. Die letzte Aktualisierung erfolgte 2019. Die Leitlinien, die die Relevanz von unterschiedlichen Erkrankungen für die gesundheitliche Eignung zum Lenken eines Kfz erfassen, sollen für Amtsärzt:innen als Unterstützung bei verkehrsrelevanten Entscheidungen dienen und mehr Klarheit für die betroffenen Führerscheinwerber bringen. Auszug aus den einleitenden Bemerkungen dieser Leitlinien zur Ausgangslage vor Erstellung: „Zahlreiche Fälle – vor allem im Zusammenhang mit Diabetes und Hypertonie – haben in der Vergangenheit gezeigt, dass den behördlich verfügten Befristungen von Lenkberechtigungen amtsärztliche Gutachten zu Grunde gelegen sind, die im Lichte der Judikatur des Verwaltungsgerichtshofes als nicht schlüssig, bzw. als unzureichend für die betreffende Befristung anzusehen waren.“

Im Kapitel „Zuckerkrankheit“ werden unabhängig vom Diabetestyp zwei Möglichkeiten der Diabetestherapie berücksichtigt und unterschiedlich bewertet.

**Möglichkeit 1: Bei Menschen mit Diabetes, die mit Medikamenten, die keine Hypoglykämien verursachen können, behandelt werden, ist keine Befristung vorgesehen, aber abhängig von den Umständen des Einzelfalls möglich.**

**Möglichkeit 2: Bei Menschen mit Diabetes, die mit Medikamenten wie Insulin, Sulfonylharnstoffen oder Gliniden behandelt werden, die schwere Hypoglykämien verursachen können, ist in Abständen von jeweils maximal 5 Jahren eine amtsärztliche Untersuchung einschließlich der Vorlage einer fachärztlichen Stellungnahme erforderlich.** Bei Lenkberechtigungen der Gruppe 2 wird das Untersuchungsintervall auf maximal 3 Jahre reduziert.

In den genannten Leitlinien werden auch andere Erkrankungen berücksichtigt, die Einfluss auf die gesundheitliche Eignung zum Lenken eines Kfz haben können, wie Herz-Kreislauf-Erkrankungen, Neuropathie, Nephropathie und andere.

Die **„Driver and Vehicle Licensing Agency“ (DVLA) in Großbritannien hat Empfehlungen für Menschen mit insulinpflichtigen Diabetes übersichtlich zusammengefasst** [40]. Allerdings wird zur Gewährleistung der Fahrsicherheit wie in vielen anderen Ländern auch gefordert, dass zusätzlich Untersuchungen hinsichtlich Augen- und Nervenschädigung, sowie Erhebung eines kardiovaskulären Risikoprofils in regelmäßigen Abständen erfolgen müssen.

Als Grundlage für die Vorgaben der EU-Führerscheinrichtlinie zu Diabetes diente der Bericht „**Diabetes and Driving in Europe; a Report of the Second European Working Group on Diabetes and Driving, an advisory board to the Driving Licence Committee of the European Union**“ [15]. Die Empfehlungen wurden in der EU-Führerscheinrichtlinie umgesetzt und waren damit auch Grundlage für die österreichische Regelung in § 11 FSG-GV.

Relativ neu ist die **Leitlinie der deutschen Diabetesgesellschaft (DDG): S2e-Leitlinie Diabetes und Straßenverkehr 1. Auflage 2017; AWMF-Register-Nr. 057-026 **[41]. Diese im Jahr 2017 erstellte Leitlinie ist sehr ausführlich gehalten, basiert auf wissenschaftlichen Daten und spricht sowohl akute verkehrsrelevante Komplikationen des Diabetes mellitus, als auch Folgeschäden, sowie rechtliche Aspekte an. Diese S2e-Leitlinie wurde auf Initiative der DDG mit anderen Fachgesellschaften und Verbänden auf der Grundlage sämtlich verfügbarer wissenschaftlicher Evidenz erstellt. Die Leitlinie ist auf der Homepage der deutschen Diabetesgesellschaft abrufbar.

www.deutsche-diabetes-gesellschaft.gesellschaft.de/leitlinien/evidenzbasierte-leitlinien.html

Darüber hinaus wurde im Jahr 2019 eine **„Patientenleitlinie Diabetes und Straßenverkehr“** verfasst (1. Auflage, Version 1 vom 06.11.2019, gültig bis November 2022) die auf der wissenschaftlichen Leitlinie basiert und sich in erster Linie an Menschen mit Diabetes mellitus richtet [42].

Auch die **schweizerische Gesellschaft für Endokrinologie und Diabetologie **hat im Jahr 2017** Richtlinien bezüglich Fahreignung und Fahrfähigkeit bei Diabetes mellitus** publiziert [43]. Auszug: „Beim Vorliegen eines Diabetes mellitus können akut auftretende oder auch langfristig bestehende Einschränkungen Einfluss auf das sichere Lenken eines Motorfahrzeuges haben, wie beispielsweise das Auftreten einer Unterzuckerung, ein deutlich überhöhter Blutzuckerspiegel oder ein vermindertes Sehvermögen als Folgekomplikation des Diabetes. Daher bestehen in der Schweiz wie auch in allen übrigen europäischen Ländern gesetzliche Regelungen bezüglich Diabetes und Verkehrsteilnahme.“ Der Fokus dieser Leitlinie liegt auf dem individuellen Risiko für Hypoglykämien, welches in drei Stufen eingeteilt wird: Hohes Hypoglykämierisiko, erhöhtes Hypoglykämierisiko und niedriges Hypoglykämierisiko. Hohes Hypoglykämierisiko ist definiert durch Vorkommen einer schweren Hypoglykämie in den letzten 2 Jahren oder Diagnose einer Hypoglykämiewahrnehmungsstörung. In der Richtlinie wird für die Diagnostik der Hypoglykämiewahrnehmungsstörung die Anwendung des Clarke Scores (1994) vorgeschlagen [23]. Die Frage „Wie häufig haben Sie Symptome einer Hypoglykämie, wenn der Blutzucker unter 54 mg/dl abfällt?“ wird auf einer Skala von 1–7 (1 = immer, 7 = nie) bewertet. Ein Wert von ≥ 4 (50 % oder weniger) ergibt die Verdachtsdiagnose einer verminderten Hypoglykämiewahrnehmung. Die Leitlinie fordert weiters die Dokumentation von Folgeschäden des Diabetes mellitus wie Retinopathie, Neuropathie, Nephropathie und Herz-Kreislauferkrankungen.

## Fachärztliche Stellungnahme, ärztliches Gutachten und Befristung des Führerscheines

In **§ 1 FSG-GV **wird zwischen ärztlichem Gutachten und fachärztlicher Stellungnahme unterschieden.

Bei der Erstellung eines ärztlichen Gutachtens oder einer fachärztlichen Stellungnahme als Grundlage für die Entscheidung der Behörde, sind eine Anamnese und eine körperliche Untersuchung durchzuführen. Erforderlichenfalls ist eine fachärztliche Stellungnahme einzuholen, einschließlich der Erhebung von Laborwerten sowie Befundeinholungen zu Spätfolgen und Begleiterkrankungen. Das ärztliche Gutachten bewertet die gesundheitliche Eignung zum Lenken eines Kfz. Dabei spielen die Stabilität der Stoffwechseleinstellung, die Art der Diabetestherapie, bereits eingetretene Spätfolgen und das sichere rechtzeitige Erkennen und Behandeln von Hypoglykämien eine zentrale Rolle.

Die Indikationsstellung für das Einholen eines ärztlichen Gutachtens unter Einbeziehung einer fachärztlichen Stellungnahme obliegt in Österreich der Behörde, Grund dafür sind Zweifel an der Fahrtauglichkeit. Das Einholen einer fachärztlichen Stellungnahme ist bei Diabetes mellitus nach Ablauf der Befristung allerdings gesetzlich immer vorgeschrieben (§ 11 Abs 1 FSG-GV).

**Ein ärztliches Gutachten** ist ein von einer Amtsärztin/einem Amtsarzt oder von einer/einem gemäß § 34 FSG bestellter/em sachverständigen Ärztin für Allgemeinmedizin erstelltes Gutachten. Sind besondere Befunde wie eine fachärztliche Stellungnahme erforderlich, ist das Gutachten jedenfalls vom Amtsarzt zu verfassen (§ 8 Abs 2 FSG). Ein von der Behörde angefordertes amtsärztliches Gutachten dient als Entscheidungsgrundlage und wird herangezogen, wenn Unklarheiten bezüglich Bewertung der Fahreignung bestehen.

**Eine fachärztliche Stellungnahme **wird vom Amtsarzt angefordert und ist von einem Facharzt des entsprechenden Sonderfaches abzugeben (Innere Medizin, Innere Medizin mit Endokrinologie und Stoffwechselerkrankungen, Innere Medizin und Endokrinologie und Diabetologie oder Modul Endokrinologie nach der neuen Ärzteausbildungsordnung 2015, Kinder- und Jugendheilkunde). In der Stellungnahme ist ein Krankheitsbild zu beschreiben und dessen Auswirkung auf das Lenken von Kraftfahrzeugen zu beurteilen. Ein standardisiertes Zuweisungsformular (siehe Anhang [32]) für die Fachärzt:innen soll den Prozess vereinfachen. Auf Grundlage dieses Zuweisungsformulars wird durch die geforderten Angaben der aktuelle Gesundheitszustand des Betroffenen dokumentiert. Im Detail werden Kenntnisse zur Abwendung von schweren Hypoglykämien, mögliche verkehrsrelevante Einschränkungen, Durchführung von Blutzuckerkontrollen und Präsenz von Spätfolgen und Begleiterkrankungen erhoben. Weiters sind eine Stellungnahme zur gesundheitlichen Eignung zum Lenken eines Kfz, sowie Angaben zu Intervallen von Kontrolluntersuchungen und Befristungen, die über das gesetzliche Ausmaß hinausgehen, erforderlich.

Die fachärztliche Stellungnahme kann prinzipiell auch von den behandelnden Ärzt:innen erstellt werden, wenn die oben angeführten Voraussetzungen erfüllt werden.

Das abschließende ärztliche Gutachten wird von Ämtsärzt:innen unter Einbeziehung der fachärztlichen Stellungnahme erstellt.

Gutachten und fachärztliche Stellungnahme sind mit nicht unerheblichen Kosten für den Fahrzeuglenker verbunden. Die Frage der Zumutbarkeit dieser zumeist wiederholten Kosten für Betroffene bleibt vorerst offen.

Befristungen sind für Menschen mit Diabetes mellitus, die mit Insulin oder anderen Medikamenten mit Hypoglykämierisiko behandelt werden, in der FSG-GV ausdrücklich geregelt. In diesen Fällen ist der Führerschein zwingend auf maximal fünf Jahre (Gruppe 1) bzw. 3 Jahre (Gruppe 2) zu befristen (§ 11 Abs. 2 und 3 FSG-GV; siehe dazu auch die zur vergleichbaren Regelung bei Epilepsie ergangene Entscheidung des Verwaltungsgerichtshofs [VwGH] vom 11.04.2022, Ra 2020/11/0222 sowie im Hinblick auf Diabetes das Landesverwaltungsgericht Niederösterreich, 06.12.2017, LVwG-AV-726/001-2017); bei kürzeren Befristungen muss die medizinische Notwendigkeit begründet werden.

In allen anderen Fällen (keine Medikamente mit Hypoglykämierisiko) ist grundsätzlich keine Befristung vorgesehen. Ist eine Befristung im Einzelfall erforderlich, muss sie begründet werden. Dabei ist allein die Tatsache, dass eine Verschlechterung des Gesundheitszustandes möglich ist bzw. nicht ausgeschlossen werden kann, laut Rechtsprechung des VwGH als Begründung zum Zweck einer Befristung nicht ausreichend. Auch die Betonung, dass es sich um eine Erkrankung mit grundsätzlich progredientem Verlauf handelt, reicht für eine Befristung nicht aus. Eine Verschlechterung des Gesundheitszustandes kann im Zeitverlauf generell bei niemandem ausgeschlossen werden. Der VwGH verlangt eine konkrete Darstellung, warum in dem speziellen Fall von einer Verschlechterung in absehbarer Zeit ausgegangen werden muss. Eine Befristung „vorsichtshalber“ ist nicht zulässig. Nach der Rechtsprechung des VwGH ist im Rahmen einer chronischen Erkrankung eine Befristung nur dann rechtmäßig, wenn nachgewiesen werden kann, dass nach dem Stand der medizinischen Wissenschaft damit gerechnet werden ***muss***, dass es im konkreten Fall zu einer die Eignung zum Lenken eines Kraftfahrzeuges ausschließenden oder einschränkenden Verschlechterung des Gesundheitszustandes kommen wird [31, 32].

Regelmäßige ärztliche Kontrolluntersuchungen innerhalb des Befristungszeitraums können in der fachärztlichen Stellungnahme vorgeschlagen und als Auflage durch die Behörde vorgeschrieben werden (§ 1 Z 5 FSG-GV). Bei Menschen mit Diabetes, die mit Medikamenten mit Hypoglykämierisiko behandelt werden, sind Kontrolluntersuchungen verpflichtend vorzuschreiben (§ 11 Abs. 2 und 3 FSG-GV). Seit 01.10.2011 (5. FSG-GV-Novelle, BGBl II 2011/280) dürfen Kontrolluntersuchungen außerdem niemals alleine, sondern **immer nur in Verbindung mit einer Befristung **der Lenkberechtigung und einer amtsärztlichen Nachuntersuchung bei Ablauf dieser Befristung verfügt werden (§ 2 Abs. 1 letzter Satz FSG-GV).

## Konklusion

Sicherheit im Straßenverkehr ist für Menschen mit Diabetes mellitus an mehrere Voraussetzungen geknüpft. Bei Therapie mit Medikamenten mit Hypoglykämierisiko sind regelmäßige Selbstkontrollen der Glukosewerte und die Absolvierung einer strukturierten Diabetesschulung für eine stabile Stoffwechselsituation von großer Bedeutung. Im Rahmen dieser Schulungen ist eine ausführliche Information über verkehrsbezogene Aspekte wie Voraussetzungen für die gesundheitliche Eignung ein Kfz zu lenken, sowie über Verhaltensmaßnahmen, die sich auf das sichere Lenken eines Kfz auswirken, erforderlich. Ebenso sind Kenntnisse über mögliche vorübergehende Einschränkungen der Fahrtauglichkeit zu vermitteln, wie z. B. im Zuge einer Neueinstellung oder einer Umstellung der bisherigen Therapie. Die schriftliche Dokumentation dieser Maßnahmen in der Patientenakte obliegt dem behandelnden Arzt.

Das Verhindern von schweren Hypoglykämien ist an folgende Anforderungen gebunden:

1. Mitführen eines Blutzuckermessgerätes oder Tragen eines Glukosesensors.
2. Messen des Blutzuckers oder Überprüfen der Sensorglukose vor Antritt einer Autofahrt.
3. Wenn der Zuckerwert < 90 mg/dl liegt, vor Fahrtantritt Kohlenhydrate zu sich nehmen.
4. Wenn der Zuckerwert < 70 mg/dl liegt, oder Symptome der Hypoglykämie auftreten, STOP Autofahrt. Zufuhr von Kohlehydraten, anschließend Glukosemessung wiederholen. Die Autofahrt erst wieder aufnehmen, wenn der Glukosewert ≥ 90 mg/dl liegt und Symptome der Hypoglykämie vollständig aufgelöst sind. In der Regel nach 15–30 min.
5. Mitführen von rasch resorbierbaren Kohlehydraten in unmittelbarer Reichweite.
6. Bei Umstellung des Behandlungsschemas ist besondere Vorsicht geboten, eventuell bei instabiler Stoffwechsellage vorübergehend Aussetzen des Lenkens von Kfz.
7. Vorsicht nach sportlicher Betätigung, eventuell häufigere Glukosemessungen.
8. Während einer längeren Autofahrt regelmäßige Messpausen zur Kontrolle der Blutglukose oder Kontrolle der Sensorglukose (2- bis 3‑Stunden Intervalle). Mahlzeitenpausen einlegen.
9. Kein Alkohol vor und während Autofahrten.

Bei Auftreten von schweren Hypoglykämien sind entsprechende Maßnahmen wie Absolvierung eines Hypogkyämiewahrnehmungstrainings, Verordnung eines kontinuierlichen Glukosemonitorings mit Hypoglykämiewarnfunktion oder einer sensorgestützten Insulinpumpentherapie zu treffen. Auch die Umstellung des Therapieschemas auf Medikamente ohne bzw. mit niedrigerem Hypoglykämiepotential kann notwendig sein, um die Fahrtauglichkeit wiederherzustellen. In der Folge sollte nach einem Ereignis mit schwerer Hypoglykämie eine Kontrolle innerhalb eines Jahres erfolgen, um die Umsetzung Hypoglykämie-vermeidender Maßnahmen und die Therapieadhärenz zu überprüfen.

Bei Menschen mit Diabetes mellitus, die mit Medikamenten ohne Hypoglykämierisiko behandelt werden, die über eine stabile Stoffwechsellage verfügen und bei denen keine schweren Folgeschäden des Diabetes mellitus bzw Begleiterkrankungen vorliegen, ist grundsätzlich keine Befristung vorzusehen.

Bei Menschen mit Diabetes mellitus, die mit Medikamenten mit Hypoglykämierisiko behandelt werden, die über eine stabile Stoffwechsellage verfügen, ausreichend geschult sind, um Hypoglykämien zu erkennen und zu behandeln (ggf. unter Einsatz eines Glukosesensors), und bei denen keine schweren Folgeschäden des Diabetes mellitus bzw. Begleiterkrankungen vorliegen, ist entsprechend der gesetzlichen Vorgabe (Kfz Gruppe 1) grundsätzlich eine Befristung auf 5 Jahre vorzusehen.

Das Screening auf Spätfolgen des Diabetes mellitus soll durch die Betroffenen unabhängig von einer Lenkberechtigung in Eigenverantwortung regelmäßig veranlasst werden. Bei fortgeschrittenen Spätfolgen mit verkehrsrelevanten Auswirkungen können auf Grundlage einer Stellungnahme einschlägiger Fachärzt:innen je nach betroffenem Organ gezielte Auflagen wie z. B. Kontrolluntersuchungen in bestimmten Abständen erteilt werden. Auch die Dauer einer Befristung kann dadurch beeinflusst werden.

Zu bedenken ist, dass im Rahmen einer Entziehung der Lenkberechtigung sowie bei Befristungen und Auflagen auch sozialmedizinische Aspekte eine Rolle spielen, ebenso sind ungünstige Auswirkungen auf das Berufsleben möglich.

Die Entziehung der Lenkberechtigung ist für die meisten Menschen ein einschneidendes und in vielerlei Hinsicht herausforderndes Ereignis mit negativen Auswirkungen auf viele Bereiche im Alltag der Betroffenen. Daher sollte die Entziehung der Lenkberechtigung bei Menschen mit Diabetes mit einer ausführlichen Aufklärung über die Möglichkeiten zur Wiedererlangung kombiniert sein. Zu diesen Möglichkeiten gehören z. B. Absolvierung einer intensiven Schulung zur Hypoglykämiewahrnehmung, Einsatz von Glukosesensoren mit Hypoglykämiewarnfunktion, Umstellung der Diabetestherapie (siehe Kapitel Maßnahmen zur Abwendung von schweren Hypoglykämien), oder auch Kontaktaufnahme mit Selbsthilfegruppen zum Erfahrungsaustausch mit Gleichgesinnten.

## Interessenkonflikt

H. Abrahamian gibt an, dass für sie persönlich kein Interessenkonflikt vorliegt. B. Salamon gibt an, dass für sie persönlich kein Interessenkonflikt vorliegt. A. Bräuer gibt an, dass für ihn persönlich kein Interessenkonflikt vorliegt. C. Schelkshorn gibt an, dass für ihn persönlich kein Interessenkonflikt vorliegt. A. Lahnsteiner gibt an, dass für sie persönlich kein Interessenkonflikt vorliegt. L. Stechemesser gibt an, dass für ihn persönlich kein Interessenkonflikt vorliegt. G. Köhler gibt an, dass für ihn persönlich kein Interessenkonflikt vorliegt. M. Clodi gibt an, dass für ihn persönlich kein Interessenkonflikt vorliegt.
